# Report of Deaths in Buffalo for the Month of September, 1855

**Published:** 1855-11

**Authors:** J. Root

**Affiliations:** Health Physician


ART. XIV.—*Report of Deaths in Buffalo for the month*

| DISEASES. | Males. | Females. | Total. | AGE. Under 1 year. Male. | Female. | 1 to 2 years. Male. | Female. |
|---|---|---|---|---|---|---|---|
| Abdominal Hæmorrhage, | .... | 1 | 1 | .. | .. | .. | .. |
| Accident, | 2 | .... | 2 | .. | .. | .. | .. |
| Atrophia, | 2 | .... | 2 | 2 | .. | .. | .. |
| Anasarca, | 1 | .... | 1 | .. | .. | .. | .. |
| Abscess of Lungs, | 1 | .... | 1 | .. | .. | .. | .. |
| Burned, | .... | 2 | 2 | .. | .. | .. | .. |
| Convulsions, | 11 | 7 | 18 | 7 | 7 | 4 | .. |
| Cholera Infantum, | 6 | 7 | 13 | 4 | 3 | 2 | 4 |
| " Morbus, | 1 | .... | 1 | 1 | .. | .. | .. |
| Cholera, | 2 | 1 | 3 | .. | .. | .. | .. |
| Congestion of Brain, | 1 | 2 | 3 | .. | .. | .. | .. |
| " of Lungs, | 1 | .... | 1 | .. | .. | .. | .. |
| Consumption, | 13 | 14 | 27 | 2 | 1 | 2 | 3 |
| Child Bed, | .... | 2 | 2 | .. | .. | .. | .. |
| Croup, | 1 | .... | 1 | .. | .. | .. | .. |
| Colic, | .... | 1 | 1 | .. | 1 | .. | .. |
| Cancrum Oris, | 1 | 1 | 2 | .. | .. | 1 | .. |
| Dentition, | 2 | 7 | 9 | 2 | 3 | .. | 3 |
| Diarrhœa (summer complaint), | 39 | 27 | 66 | 2 | 12 | 11 | 8 |
| Dysentery, | 4 | 7 | 11 | 1 | 3 | 1 | 1 |
| Drowned, | 4 | .... | 4 | .. | .. | .. | .. |
| Debility, | 3 | 4 | 7 | 3 | 4 | .. | .. |
| Dropsy, | 1 | 2 | 3 | .. | .. | .. | .. |
| " of Brain, | .... | 1 | 1 | .. | .. | .. | 1 |
| Disease of Stomach, | .... | 1 | 1 | .. | .. | .. | .. |
| Epilepsy, | 1 | .... | 1 | 1 | .. | .. | .. |
| Fever, | 4 | 1 | 5 | 2 | .. | .. | .. |
| " Bilious, | .... | 1 | 1 | .. | .. | .. | .. |
| " Typhus, | 1 | 2 | 3 | .. | .. | .. | .. |
| " Typhoid, | 4 | 1 | 5 | .. | .. | .. | .. |
| " Scarlet, | 5 | 1 | 6 | 1 | .. | .. | .. |
| " Intermittent Malignant, | 1 | .... | 1 | .. | .. | .. | .. |
| "Febris Nervosa," | 1 | .... | 1 | .. | .. | .. | .. |
| Gangrene, succeeding circumcision, | 1 | .... | 1 | 1 | .. | .. | .. |
| Hydrocephalus, | 7 | 1 | 8 | 4 | .. | 2 | 2 |
| Inflammation of Bowels, | 2 | 1 | 3 | 1 | 1 | 1 | .. |
| Inflammation of Brain, | .... | 1 | 1 | .. | .. | .. | 1 |
| Inflammation of Womb, | .... | 1 | 1 | .. | .. | .. | .. |
| Laryngitis, | 1 | .... | 1 | .. | .. | .. | .. |
| Marasmus, | 1 | .... | 1 | .. | .. | 1 | .. |
| Old Age, | 2 | 1 | 3 | .. | .. | .. | .. |
| Opium Eating, | .... | 1 | 1 | .. | .. | .. | .. |
| Pneumonia, | 2 | 1 | 3 | 1 | .. | .. | 1 |
| Premature Birth, | 1 | .... | 1 | 1 | .. | .. | .. |
| Still-born, | 3 | 3 | 6 | 3 | 3 | .. | .. |
| Sea Sickness, | 1 | .... | 1 | .. | .. | 1 | .. |
| Typhoid Pneumonia, | 1 | 1 | 2 | .. | .. | .. | .. |
| Unknown, | 10 | 7 | 17 | 7 | 4 | .. | 1 |
| Whooping Cough, | 6 | 4 | 10 | 3 | 1 | .. | 2 |
| | .... | .... | .... | .. | .. | .. | .. |
| Totals, | 151 | 115 | 266 | 70 | 43 | 26 | 27 |

NATIVITIES.—American, 113. German, 91. English, 3. Irish, 24. French, 4.

*of September*, 1855. By J. Root, M. D., Health Physician.

AGE.

| 2—5 | | 5—10 | | 10—15 | | 15—20 | | 20—25 | | 25—30 | | 30—40 | | 40—50 | | 50—60 | | 60—70 | | 70—80 | | 80—90 | | 90—100 | | 100 and upward. | | Not given. | |
|---|---|---|---|---|---|---|---|---|---|---|---|---|---|---|---|---|---|---|---|---|---|---|---|---|---|---|---|---|---|
| Male. | Female. | Male. | Female. | Male. | Female. | Male. | Female. | Male. | Female. | Male. | Female. | Male. | Female. | Male. | Female. | Male. | Female. | Male. | Female. | Male. | Female. | Male. | Female. | Male. | Female. | Male. | Female. | Male. | Female. |
| .. | .. | .. | .. | .. | .. | .. | .. | .. | 1 | .. | .. | .. | .. | .. | .. | .. | .. | .. | .. | .. | .. | .. | .. | .. | .. | .. | .. | .. | .. |
| 1 | .. | .. | .. | .. | .. | .. | .. | .. | .. | .. | .. | .. | .. | .. | .. | 1 | .. | .. | .. | .. | .. | .. | .. | .. | .. | .. | .. | .. | .. |
| .. | .. | .. | .. | .. | .. | .. | .. | .. | .. | .. | .. | .. | .. | .. | .. | .. | .. | .. | .. | .. | .. | .. | .. | .. | .. | .. | .. | .. | .. |
| 1 | .. | .. | .. | .. | .. | .. | .. | .. | .. | .. | .. | .. | .. | .. | .. | .. | .. | .. | .. | .. | .. | .. | .. | .. | .. | .. | .. | .. | .. |
| .. | .. | .. | .. | .. | .. | .. | .. | .. | .. | .. | .. | .. | .. | .. | .. | .. | .. | .. | .. | .. | .. | .. | .. | .. | .. | .. | .. | 1 | .. |
| .. | .. | .. | 2 | .. | .. | .. | .. | .. | .. | .. | .. | .. | .. | .. | .. | .. | .. | .. | .. | .. | .. | .. | .. | .. | .. | .. | .. | .. | .. |
| .. | .. | .. | .. | .. | .. | .. | .. | .. | .. | .. | .. | .. | .. | .. | .. | .. | .. | .. | .. | .. | .. | .. | .. | .. | .. | .. | .. | .. | .. |
| .. | .. | .. | .. | .. | .. | .. | .. | .. | .. | .. | .. | .. | .. | .. | .. | .. | .. | .. | .. | .. | .. | .. | .. | .. | .. | .. | .. | .. | .. |
| .. | .. | .. | .. | .. | .. | .. | .. | .. | .. | .. | .. | .. | .. | .. | .. | .. | .. | .. | .. | .. | .. | .. | .. | .. | .. | .. | .. | .. | .. |
| .. | .. | .. | .. | .. | .. | 1 | .. | .. | 1 | .. | .. | 1 | .. | .. | .. | .. | .. | .. | .. | .. | .. | .. | .. | .. | .. | .. | .. | .. | .. |
| .. | 1 | .. | .. | .. | .. | 1 | .. | .. | .. | .. | .. | .. | .. | .. | 1 | .. | .. | .. | .. | .. | .. | .. | .. | .. | .. | .. | .. | .. | .. |
| .. | .. | .. | .. | .. | .. | .. | .. | .. | .. | .. | .. | .. | .. | .. | .. | 1 | .. | .. | .. | .. | .. | .. | .. | .. | .. | .. | .. | .. | .. |
| 1 | 1 | .. | 1 | .. | 1 | 1 | 1 | 1 | 1 | .. | 1 | 1 | 1 | 2 | 2 | 1 | .. | 2 | 1 | .. | .. | .. | .. | .. | .. | .. | .. | .. | .. |
| .. | .. | .. | .. | .. | .. | .. | .. | .. | .. | .. | .. | .. | .. | .. | 1 | .. | .. | .. | .. | .. | .. | .. | .. | .. | .. | .. | .. | .. | 1 |
| 1 | .. | .. | .. | .. | .. | .. | .. | .. | .. | .. | .. | .. | .. | .. | .. | .. | .. | .. | .. | .. | .. | .. | .. | .. | .. | .. | .. | .. | .. |
| .. | .. | .. | .. | .. | .. | .. | .. | .. | .. | .. | .. | .. | .. | .. | .. | .. | .. | .. | .. | .. | .. | .. | .. | .. | .. | .. | .. | .. | .. |
| .. | 1 | .. | .. | .. | .. | .. | .. | .. | .. | .. | .. | .. | .. | .. | .. | .. | .. | .. | .. | .. | .. | .. | .. | .. | .. | .. | .. | .. | .. |
| .. | 1 | .. | .. | .. | .. | .. | .. | .. | .. | .. | .. | .. | .. | .. | .. | .. | .. | .. | .. | .. | .. | .. | .. | .. | .. | .. | .. | .. | .. |
| 5 | 4 | .. | 1 | .. | .. | .. | .. | .. | .. | .. | 1 | .. | .. | .. | .. | .. | 1 | .. | .. | .. | .. | .. | .. | .. | .. | .. | .. | .. | .. |
| .. | 1 | .. | 1 | .. | .. | .. | .. | .. | .. | .. | 1 | .. | 1 | 1 | .. | .. | .. | .. | .. | .. | .. | .. | .. | .. | .. | .. | .. | .. | .. |
| .. | .. | .. | 1 | .. | .. | 1 | .. | 1 | .. | 1 | .. | .. | .. | .. | .. | .. | .. | .. | .. | .. | .. | .. | .. | .. | .. | .. | .. | .. | .. |
| .. | .. | .. | .. | 1 | .. | .. | .. | .. | .. | .. | .. | .. | .. | .. | .. | .. | .. | .. | .. | .. | .. | .. | .. | .. | .. | .. | .. | .. | .. |
| .. | .. | .. | .. | .. | .. | .. | .. | .. | .. | .. | .. | .. | .. | .. | 1 | .. | .. | 1 | 1 | .. | .. | .. | .. | .. | .. | .. | .. | .. | .. |
| .. | .. | .. | .. | .. | .. | .. | .. | .. | .. | .. | .. | .. | .. | .. | .. | .. | .. | .. | .. | .. | .. | .. | .. | .. | .. | .. | .. | .. | .. |
| .. | .. | .. | .. | .. | .. | .. | .. | .. | .. | .. | .. | .. | .. | .. | .. | .. | 1 | .. | .. | .. | .. | .. | .. | .. | .. | .. | .. | .. | .. |
| .. | .. | .. | .. | .. | .. | .. | .. | .. | .. | .. | .. | .. | .. | .. | .. | .. | .. | .. | .. | .. | .. | .. | .. | .. | .. | .. | .. | .. | .. |
| .. | .. | 1 | .. | .. | .. | .. | .. | .. | .. | .. | .. | 1 | .. | .. | .. | .. | 1 | .. | .. | .. | .. | .. | .. | .. | .. | .. | .. | .. | .. |
| .. | .. | .. | .. | .. | .. | .. | .. | .. | .. | .. | .. | .. | .. | .. | .. | .. | .. | .. | 1 | .. | .. | .. | .. | .. | .. | .. | .. | .. | .. |
| .. | .. | .. | .. | .. | .. | .. | .. | 1 | 1 | .. | .. | .. | 1 | .. | .. | .. | .. | .. | .. | .. | .. | .. | .. | .. | .. | .. | .. | .. | .. |
| .. | .. | .. | .. | .. | .. | 1 | .. | 1 | 1 | 1 | .. | 1 | .. | .. | .. | .. | .. | .. | .. | .. | .. | .. | .. | .. | .. | .. | .. | .. | .. |
| 1 | 1 | 3 | .. | .. | .. | .. | .. | .. | .. | .. | .. | .. | .. | .. | .. | .. | .. | .. | .. | .. | .. | .. | .. | .. | .. | .. | .. | .. | .. |
| .. | .. | .. | .. | .. | .. | 1 | .. | .. | .. | .. | .. | .. | .. | .. | .. | .. | .. | .. | .. | .. | .. | .. | .. | .. | .. | .. | .. | .. | .. |
| .. | .. | .. | .. | .. | .. | .. | .. | .. | .. | .. | .. | .. | .. | .. | .. | 1 | .. | .. | .. | .. | .. | .. | .. | .. | .. | .. | .. | .. | .. |
| .. | .. | .. | .. | .. | .. | .. | .. | .. | .. | .. | .. | .. | .. | .. | .. | .. | .. | .. | .. | .. | .. | .. | .. | .. | .. | .. | .. | .. | .. |
| .. | .. | .. | .. | .. | .. | .. | .. | .. | .. | .. | .. | .. | .. | .. | .. | .. | .. | .. | .. | .. | .. | .. | .. | .. | .. | .. | .. | .. | .. |
| .. | .. | .. | .. | .. | .. | .. | .. | .. | .. | .. | .. | .. | .. | .. | .. | .. | .. | .. | .. | .. | .. | .. | .. | .. | .. | .. | .. | .. | .. |
| .. | .. | .. | .. | .. | .. | .. | .. | .. | .. | .. | .. | .. | .. | .. | .. | .. | .. | .. | .. | .. | .. | .. | .. | .. | .. | .. | .. | .. | .. |
| .. | .. | .. | .. | .. | .. | .. | .. | .. | .. | .. | .. | .. | 1 | .. | .. | .. | .. | .. | .. | .. | .. | .. | .. | .. | .. | .. | .. | .. | .. |
| 1 | .. | .. | .. | .. | .. | .. | .. | .. | .. | .. | .. | .. | .. | .. | .. | .. | .. | .. | .. | .. | .. | .. | .. | .. | .. | .. | .. | .. | .. |
| .. | .. | .. | .. | .. | .. | .. | .. | .. | .. | .. | .. | .. | .. | .. | .. | .. | .. | .. | .. | .. | .. | .. | .. | .. | .. | .. | .. | .. | .. |
| .. | .. | .. | .. | .. | .. | .. | .. | .. | .. | .. | .. | .. | .. | .. | .. | .. | 1 | 1 | .. | 1 | .. | .. | .. | .. | .. | .. | .. | .. | .. |
| .. | .. | .. | .. | .. | .. | .. | .. | .. | .. | .. | .. | .. | .. | .. | .. | .. | .. | .. | .. | .. | 1 | .. | .. | .. | .. | .. | .. | .. | .. |
| .. | .. | 1 | .. | .. | .. | .. | .. | .. | .. | .. | .. | .. | .. | .. | .. | .. | .. | .. | .. | .. | .. | .. | .. | .. | .. | .. | .. | .. | .. |
| .. | .. | .. | .. | .. | .. | .. | .. | .. | .. | .. | .. | .. | .. | .. | .. | .. | .. | .. | .. | .. | .. | .. | .. | .. | .. | .. | .. | .. | .. |
| .. | .. | .. | .. | .. | .. | .. | .. | .. | .. | .. | .. | .. | .. | .. | .. | .. | .. | .. | .. | .. | .. | .. | .. | .. | .. | .. | .. | .. | .. |
| .. | .. | .. | .. | .. | .. | .. | .. | .. | .. | .. | .. | .. | .. | .. | .. | .. | .. | .. | .. | .. | .. | .. | .. | .. | .. | .. | .. | .. | .. |
| .. | 1 | 1 | .. | .. | .. | .. | .. | .. | .. | .. | .. | .. | .. | .. | .. | .. | .. | .. | .. | .. | .. | .. | .. | .. | .. | .. | .. | .. | .. |
| .. | 1 | .. | 1 | .. | .. | .. | .. | .. | 1 | .. | .. | .. | .. | .. | .. | .. | .. | 1 | .. | .. | .. | .. | .. | .. | .. | .. | .. | 1 | .. |
| 2 | .. | .. | 2 | .. | .. | .. | .. | .. | .. | .. | .. | .. | .. | .. | .. | .. | .. | .. | .. | .. | .. | .. | .. | .. | .. | .. | .. | .. | .. |
| .. | .. | .. | .. | .. | .. | .. | .. | .. | .. | .. | .. | .. | .. | .. | .. | .. | .. | .. | .. | .. | .. | .. | .. | .. | .. | .. | .. | .. | .. |
| .. | .. | .. | .. | .. | .. | .. | .. | .. | .. | .. | .. | .. | .. | .. | .. | .. | .. | .. | .. | .. | .. | .. | .. | .. | .. | .. | .. | .. | .. |
| 13 | 12 | 6 | 8 | 1 | 1 | 6 | 1 | 4 | 6 | 2 | 3 | 4 | 4 | 3 | 5 | 4 | 4 | 5 | 3 | 1 | 1 | 0 | 0 | 0 | 0 | 0 | 0 | 2 | 1 |

Scotch, 1. Hollander, 2. Canadian, 2. Col'd, 5. Country not given, 22.—Total, 266.

